# Thoracentesis

**Published:** 1870-10

**Authors:** 


### Thoracentesis.

Mr. Berkeley Hill thinks operative procedure for the cure of pleuritic effusion has not received sufficient attention. In an article in the *British Medical Journal* he states that the following points demand consideration: (quoted in the *Medical Archives*).

1. The removal of fluid from the pleura need hardly ever cause much danger or suffering. 2. Whenever effusion is copious, it is prudent to withdraw it to relieve dyspnœa and ward off a sudden fatal termination. 3. The usual mode of leaving chronic effusions to natural resorption may be advantageously replaced by tapping whenever the condition of the patient is stationary, and the pyrexia abated; the longer the fluid has existed the more urgent becomes the need of tapping, to enable the lung to expand before it has lost its power of doing so. 4. After tapping serous effusions the wound should always be closed, at least until repetition of the evacuations had shown that cure would not be so effected; then, to tapping, injection of iodine or other irritants should be added, but in these cases the admission of air should be scrupulously avoided. 5. Where the fluid is purulent, the admission of air is immaterial, provided a free and continuous exit is maintained for the pus; and this free drainage is a cardinal point in the cure. One case illustrates the benefit from continued drainage by a tube passed across the chest through two openings; the second, the benefit which even cases of long standing empyema, where the cavity will not contract, may obtain from frequent washing out with water or other injections. The third showed how readily a serous effusion may be absorbed, even during the progress of rapid pulmonary phthisis, and in this case evacuation gave temporary relief of a considerable amount, though it did not cure the effusion.

